# Supplementary material for: Stability analysis of heterogeneous oligopoly games of increasing players: A computational approach
Source: PLoS One. 2025 Nov 3;20(11):e0335516. doi: 10.1371/journal.pone.0335516 (PMC12582497; doi:10.1371/journal.pone.0335516)
Supplement: Attachment — Submitted filename: reply.pdf [file pone.0335516.s001.pdf]

Journal Requirements:

When submitting your revision, we need you to address these additional requirements.

1. Please ensure that your manuscript meets PLOS ONE's style requirements, including those for file naming. The PLOS ONE style templates can be found at https://journals.plos.org/plosone/s/file?id=wjVg/PLOSOne_formatting_sample_main_body.pdf and https://journals.plos.org/plosone/s/file?id=ba62/PLOSOne_formatting_sample_title_authors_affiliations.pdf

Reply: We are appreciate for this comment. The manuscript has been carefully revised to meet PLOS ONE's style requirements.

2. We noticed you have some minor occurrence of overlapping text with the following previous publication(s), which needs to be addressed:

https://www.mdpi.com/2504-3110/6/8/459?

In your revision ensure you cite all your sources (including your own works), and quote or rephrase any duplicated text outside the methods section. Further consideration is dependent on these concerns being addressed.

Reply: In the updated version, we have reduced the occurrence of overlapping text with our previous paper: https://www.mdpi.com/2504-3110/6/8/459

3. Thank you for stating the following in the Acknowledgments Section of your manuscript:

The authors are grateful to the anonymous referees for their helpful comments. The first author is partially supported by Key Laboratory of Interdisciplinary Research of Computation and Economics (Shanghai University of Finance and Economics) and Innovation Team Project of Guangdong Colleges and Universities (Grant No. 2024WCXTD019). An earlier and preliminary draft of this article can be found at: https://arxiv.org/pdf/ 2112.13844.

We note that you have provided funding information that is not currently declared in your Funding Statement. However, funding information should not appear in the Acknowledgments section or other areas of your manuscript. We will only publish funding information present in the Funding Statement section of the online submission form.

Please remove any funding-related text from the manuscript and let us know how you would like to update your Funding Statement. Currently, your Funding Statement reads as follows:

The second author is partially supported by Key Laboratory of Interdisciplinary Research of Computation and Economics (Shanghai University of Finance and Economics) and Innovation Team Project of Guangdong Colleges and Universities (Grant No. 2024WCXTD019).

Please include your amended statements within your cover letter; we will change the online submission form on your behalf.

Reply: Thanks so much for pointing out this. In the updated version, the "Funding Information" section has been added. The correct information is the same as the online submission form:

"The second author is partially supported by Key Laboratory of Interdisciplinary Research of Computation and Economics (Shanghai University of Finance and Economics) and Innovation Team

Project of Guangdong Colleges and Universities (Grant No. 2024WCXTD019). The funders had no role in study design, data collection and analysis, decision to publish, or preparation of the manuscript.”

4. We note that the grant information you provided in the ‘Funding Information’ and ‘Financial Disclosure’ sections do not match.
When you resubmit, please ensure that you provide the correct grant numbers for the awards you received for your study in the ‘Funding Information’ section.

Reply: In the updated version, the “Funding Information” section has been added.

5. We note that your Data Availability Statement is currently as follows: All relevant data are within the manuscript and its Supporting Information files.

Please confirm at this time whether or not your submission contains all raw data required to replicate the results of your study. Authors must share the “minimal data set” for their submission. PLOS defines the minimal data set to consist of the data required to replicate all study findings reported in the article, as well as related metadata and methods
(https://journals.plos.org/plosone/s/data-availability#loc-minimal-data-set-definition).

For example, authors should submit the following data:

- The values behind the means, standard deviations and other measures reported;
- The values used to build graphs;
- The points extracted from images for analysis.

Authors do not need to submit their entire data set if only a portion of the data was used in the reported study.

If your submission does not contain these data, please either upload them as Supporting Information files or deposit them to a stable, public repository and provide us with the relevant URLs, DOIs, or accession numbers. For a list of recommended repositories, please see https://journals.plos.org/plosone/s/recommended-repositories.

If there are ethical or legal restrictions on sharing a de-identified data set, please explain them in detail (e.g., data contain potentially sensitive information, data are owned by a third-party organization, etc.) and who has imposed them (e.g., an ethics committee). Please also provide contact information for a data access committee, ethics committee, or other institutional body to which data requests may be sent. If data are owned by a third party, please indicate how others may request data access.

Reply: **The Python code is attached, as it may be required by the journal. It serves as supplementary data to facilitate the reproduction of our numerical simulations.**

6. Please review your reference list to ensure that it is complete and correct. If you have cited papers that have been retracted, please include the rationale for doing so in the manuscript text, or remove these

references and replace them with relevant current references. Any changes to the reference list should be mentioned in the rebuttal letter that accompanies your revised manuscript. If you need to cite a retracted article, indicate the article's retracted status in the References list and also include a citation and full reference for the retraction notice.

Reply: We have checked the reference list carefully.

Please use the space provided to explain your answers to the questions above. You may also include additional comments for the author, including concerns about dual publication, research ethics, or publication ethics. (Please upload your review as an attachment if it exceeds 20,000 characters)
Reviewer #1: This study has examined oligopolistic competitive markets with varying numbers of heterogeneous firms, focusing on the isoelastic demand and quadratic cost functions. The models considered include a range of oligopoly scenarios where the number of participating firms increases from two to five. By gradually adding more heterogeneous enterprises, new games have been created. The analytical conditions for the local stability of the Cournot-Nash equilibrium of each model under consideration have been obtained. Numerical simulations have been conducted to explore the complex dynamics, such as periodic and chaotic orbits, when the game equilibrium loses its stability. Furthermore, numerical simulations have also investigated the case of distinct cost parameters. A computational tool called the CAD method has been employed to select sample points of semi-algebraic sets. The CAD method has been applied to systematically and automatically determine the local stability conditions for the equilibrium of the models considered in this manuscript, offering more comprehensible proofs than the pencil-and-paper approach. I have the following concerns.

Reply: We really appreciate the reviewer's insightful comments on our manuscript which are greatly helpful to improve our study. In the updated version, we have revised our manuscript following the comments one by one. All the revised parts are marked in red color. Our responds to the reviewer's comment are as follows.

1. The manuscript needs extensive revision for language and grammar.

Reply: Thanks for this suggestion. In the revised version, the language has been carefully checked.

2. The manuscript’s short title in the table on the first page is the same as the complete title. It should be shorter than the original title.

Reply: Thanks. The short title has been revised.

3. The second author has two affiliations, which should be marked with different letters (b and c).

Reply: Thanks for this comment. The manuscript has been modified according to this comment.

4. All in-text mathematical expressions should be written in italics.

Reply: We are appreciate for pointing out this. The manuscript has been carefully checked.

5. After introducing an abbreviation by its complete form in its first use, all complete forms should be replaced by the abbreviation.

Reply: Thanks for this comment. In the updated version, the use of the abbreviation LMA and CAD has been revised according to this comment.

6. Several references are not explained adequately in the sixth paragraph of the introduction. Each reference should be described at least in an individual sentence.

Reply: We are appreciate for this suggestion. In the newest version, we have revised the sixth paragraph of the introduction.

7. The equations in the introduction and their associated text should be moved to section 2.

Reply: We are appreciate for this comment. We have moved these equations to the beginning of Section 3 rather than Section 2.

8. All equations should be numbered throughout the manuscript.

Reply: In the newest version, all equations have been numbered.

9. What does 'isoelastic demand function' mean, and how can it be understood that a demand function is isoelastic or not?

Reply: Thanks for this question. In the updated version, below (6), we add a short explanation:

> "Simple calculations show that the price elasticity of the market demand is exactly one."

10. At the bottom of page 8, in the equation before 'which implies that', the last term should be multiplied by '(q1(t)+q2(t+1))'. In the paragraph below Equation (9), there is an extra '(' on the right-hand side of the expression related to 'R3'. The 'CD_{GNA}' on page 20 should be corrected as 'CD_{GNAL}'. There are two '(a)' in the caption of Figure 3, and one of them should be deleted. In the partial derivatives on page 26, 'q5' in the denominator of fractions should be corrected as 'R5'. In the last paragraph of page 27, the second 'c=1' should be corrected as 'k=1'.

Reply: Thanks so much for this helpful comment. In the updated version, Eq. (18) has been modified according to the reviewer's comment. We have fixed the typo in the expression related to 'R3' on Page 18. The 'CD_{GNA}' on page 20 has been revised to 'CD_{GNAL}'. The caption of Figure 3 has been revised according to the comment. As suggested by the reviewer, 'q5' in the partial derivatives on page 26 has been changed into 'R5'. Finally, on page 27, the typo 'c=1' has been corrected to 'k=1'.

11. In all bifurcation diagrams, the x-axis should be limited to the bifurcation parameter interval, and the empty regions should be removed. Also, the bifurcation parameter should change with smaller steps to avoid discontinuities at the bifurcation points. All bifurcation parameters should be replotted.

Reply: Thanks for this suggestion. In the newest version, all one-dimensional bifurcation diagrams have been replotted.

12. All color bars should have titles, and the colors associated with chaotic and unbounded behaviors should be marked alongside different periods.

Reply: Thanks for this suggestion. We have tried to name all color bars, but the results show that the text is too crowded. Therefore, we would like to keep the original principle of naming the bars every fifth color, which didn't seem to hinder the understanding of the 2D bifurcation diagram. In addition, the color associated with chaos is yellow, whereas the one associated with unbounded trajectories is grey.

13. The ‘Acknowledgement’ and ‘Data Availability Statement’ at the end of the manuscript do not agree with those in the table at the beginning of the manuscript. They should be unified.

Reply: Thanks for pointing this out. We have updated the “Acknowledgments” and “Data Availability Statement” section.

14. The ‘Future Works’ and ‘Authors Contributions’ sections should be added to the manuscript.

Reply: The future work has been discussed in Section 8. Furthermore, the “Authors Contributions” section has been added in the updated version.

Reviewer #2: Dear Authors,
I read this manuscript and found it very useful. The authors need to consider the following comments.
1. You need to cite the number of the reference and not the year of the publication. Please the introduction part.

Reply: Thanks for pointing out this. The manuscript has been revised according to this comment.

2. In page 8, the second equation (q*_1-2cq*_2(q*_1+q*_2)=0) in the third system should be revised.

Reply: We do not fully understand this question. As we have checked carefully, the second equation in Eq. (14) is correct.

3. In Fig. 1, you considered a fixed value for k (k=1.0). What happen for other values?

Reply: Thanks for this question. Figure 1 (b) depicts the one-dimensional bifurcation diagram with respect to c. Furthermore, the effects of k and c can be found in Figure 1 (c), while the joint effects of c1 and c2 can be observed in Figure 1 (d).

4. You need to mention that (c>0) in all the equilibrium points.

Reply: We are appreciate for pointing out this. The assumption c>0 is important for the following analysis, which has been emphasized below Eq. (5):

> “where $c>0$ is a uniform parameter for all firms.”

5. The introduction section should be more informative. The relative papers are highly recommended to improve this section. I suggest some papers such as:
https://dx.doi.org/10.22436/jmcs.036.03.05, https://doi.org/10.1142/S0217984925501039, https://doi.org/10.28924/2291-8639-21-2023-131, https://doi.org/10.3390/fractalfract7050344

Reply: Thanks for this suggestion. In the updated version, the following papers have been cited:
1. Almatrafi, M. B. & Berkal, M. Bifurcation analysis and chaos control for fractional predator-prey model with Gompertz growth of prey population. Modern Physics Letters B (2024).
2. Berkal, M. & Almatrafi, M. B. Bifurcation and stability of two-dimensional activator–inhibitor model with fractional-order derivative. Fractal and Fractional 7, 344 (2023).
