# Supplementary material for: Stability analysis of heterogeneous oligopoly games of increasing players: A computational approach
Source: PLoS One. 2025 Nov 3;20(11):e0335516. doi: 10.1371/journal.pone.0335516 (PMC12582497; doi:10.1371/journal.pone.0335516)
Supplement: Attachment — Submitted filename: reply_round2.pdf [file pone.0335516.s002.pdf]

Reviewer #3: This paper investigates Cournot competition with heterogeneous firms under isoelastic demand and quadratic cost functions. By sequentially introducing additional heterogeneous firms, the authors analyze how the size of the stability region evolves as the number of firms increases. The paper makes use of the cylindrical algebraic decomposition method to rigorously derive conditions for the local stability of the Cournot–Nash equilibrium, and it complements the analytical findings with numerical simulations that highlight the emergence of periodic and chaotic dynamics once stability is lost. The additional exploration of heterogeneous cost parameters further enriches the study.

The topic is relevant, lying at the intersection of nonlinear dynamics, computational methods, and oligopoly theory. The results are both mathematically rigorous and economically insightful. I recommend acceptance, but some minor revisions are needed.

Reply: We sincerely appreciate the reviewer's insightful feedback on our manuscript, which has been extremely valuable in enhancing our study. In response to the comments, we have thoroughly revised the manuscript, addressing each point individually. All revisions are highlighted in red for easy identification. Our detailed responses to the reviewer's comments are provided below.

1. It appears that the CAD method introduced in Section 2 is not a new approach, but rather one that was developed in the last century by others (e.g., Collins and Hong). Although the authors make very good use of this method to prove their theorems (and I must admit that this proof technique is quite different from traditional approaches, which I had not seen before), I believe that the detailed description of the CAD method in Section 2 could be removed, with only appropriate references provided instead. This is because the paper is a research article rather than a textbook, and therefore it is not necessary to include a simple example to illustrate the computational procedure of the CAD method.

Reply: We sincerely appreciate the reviewer's insightful comment. Indeed, therefore it is not necessary to include a simple example to illustrate the computational procedure of the CAD method. In the revised version, we have removed Section 2. In Page 12 where the CAD method is first applied, we have added the following footnote to briefly introduce its main ideas:

"Below, theorem proofs mostly rely on the CAD method, which is a symbolic computation method. The reader will see that the introduction of the CAD method allows the deduction of stability conditions of the considered models to be conducted systematically and automatically, thus greatly simplifying the theorem proving. The CAD method is the first practical quantifier elimination algorithm, proposed by Collins [9], and is therefore also known as Collins' algorithm. This algorithm decomposes any semi-algebraic set in the n-dimensional real number space R^n into a finite number of disjoint semi-algebraic sets. All the resulting semi-algebraic sets are defined by the same set of polynomials, and the sign of the polynomials defined on each semi-algebraic set remains unchanged. The original CAD

method was not efficient enough and was later improved by Brown [8], Collins and Hong [10]}. Specifically, the CAD method can compute the cylindrical algebraic decomposition and its sample point set on R^n, such that the signs of the given polynomials are invariant in each decomposition. More details can be found in, e.g., [8-10].”

2. The language of the manuscript still requires further refinement in order to meet publication standards.

Reply: We appreciate this suggestion. In the updated version, we have carefully refined the language of the manuscript and corrected several grammatical errors.

3. The formulas in the manuscript need to be reformatted, as their current presentation appears rather disorganized.

Reply: We appreciate the reviewer for this comment. In the latest version, several formulas have been reformatted to improve the overall presentation of the paper.

4. Perhaps the authors could clarify whether the symbolic computation method they employ is entirely error-free. Thus, the results of symbolic computation can be regarded as rigorous and sufficient for establishing mathematical theorems.

Reply: Thanks for this comment. At the end of the third-to-last paragraph in Section 1, we have provided an explanation for this issue:

“It should be emphasized that this computational tool is symbolic in the sense that its results are exact and error-free, making it well-suited for discovering and proving economic theorems, as discussed in [18, 19, 21]. In comparison, numerical methods are widely used in the computation of equilibria. However, numerical methods have several shortcomings: first, numerical computation may encounter the problem of instability, which could make the results completely useless; second, most numerical algorithms only search for a single equilibrium and are nearly infeasible for detecting multiple equilibria.”

5. It is recommended that the authors discuss in the conclusion the potential applications of the CAD method in other areas of economics.

Reply: We sincerely appreciate the reviewer’s suggestion. In the second-to-last paragraph of Section 7, we have discussed the potential applications of the CAD method in other areas of economics:

“To derive the stability conditions, we employed a computational method known as CAD, which allows for the exact symbolic analysis of semi-algebraic sets. Unlike numerical approaches, the CAD method yields exact and error-free results, making it particularly suitable for proving economic theorems rigorously. We applied this method to systematically and automatically determine the local stability conditions of the models considered, offering clearer and more accessible proofs compared to the manual, pencil-and-paper approach used by Tramontana et al. [29]. Readers can see that the CAD method offers clear advantages in

analytical exploration. Beyond industrial organization theory, it also holds potential applications in algorithmic game theory and theoretical macroeconomic analysis.”
